# A mechanical Turing machine: blueprint for a biomolecular computer

**DOI:** 10.1098/rsfs.2011.0118

**Published:** 2012-03-21

**Authors:** Ehud Shapiro

**Affiliations:** Department of Computer Science and Applied Math and Department of Biological Chemistry, Weizmann Institute of Science, Rehovot 76100, Israel

**Keywords:** Turing machine, biomolecular computer, computing device

## Abstract

We describe a working mechanical device that embodies the theoretical computing machine of Alan Turing, and as such is a universal programmable computer. The device operates on three-dimensional building blocks by applying mechanical analogues of polymer elongation, cleavage and ligation, movement along a polymer, and control by molecular recognition unleashing allosteric conformational changes. Logically, the device is not more complicated than biomolecular machines of the living cell, and all its operations are part of the standard repertoire of these machines; hence, a biomolecular embodiment of the device is not infeasible. If implemented, such a biomolecular device may operate *in vivo*, interacting with its biochemical environment in a program-controlled manner. In particular, it may ‘compute’ synthetic biopolymers and release them into its environment in response to input from the environment, a capability that may have broad pharmaceutical and biological applications.

## 1. Introduction

In 1936, Turing [1] proposed a ‘pencil-and-paper’ computing device, now called the *Turing machine*, as a formalization of the notion of a procedure. Although the Turing machine is prevalent in theoretical computer science and is theoretically a universal computer, it was never realized as an actual computing device. All present day computers are based on a different architecture, the electronic computer architecture devised by von Neumann and colleagues in the 1940s [2], which uses random (logarithmic) access to stored-programs and data, as opposed to the linear sequential access employed by the universal Turing machine. In 1994, Adleman [3] showed how to compute using DNA molecules and standard molecular biology laboratory techniques. Adleman's method involves encoding combinatorial search problems with DNA sequences, and using *in vitro* selection techniques to synthesize and isolate DNA sequences that encode solutions to these problems. Subsequent works [4,5] further developed and expanded this research direction.

Adelman, apparently not being aware of the work of Bennet [6] discussed below, concluded his seminal paper by saying: ‘In the future, research in molecular biology may provide improved techniques for manipulating macromolecules. Research in chemistry may allow for the development of synthetic designer enzymes. One can imagine the eventual emergence of a general-purpose computer consisting of nothing more than a single macromolecule conjugated to a ribosome-like collection of enzymes that act on it’. Here, we attempt to advance this vision by proposing a detailed logical design for such a computer, with the ultimate goal of constructing a general-purpose programmable computer that can operate *in vivo* and interact with its biochemical environment. As the tools of molecular biology and chemistry are insufficient at present to realize this design with biomolecules, we realized it in a working mechanical implementation. This mechanical device serves as a proof-of-concept of the logical design as well as a high-level operational specification for a biomolecular implementation.

## 2. Results

**Figure 1.**
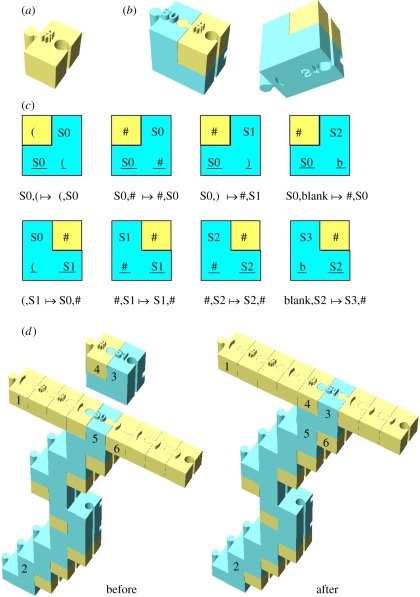
Transition molecules and operation of a *parenthesis checker* program. A *parenthesis checker* verifies that a string consisting of left and right parentheses is well-formed. For example, ‘()’, ‘(())’ and ‘(())()’ are well-formed, whereas ‘(()’, ‘)(’ and ‘()())’ are not. It operates by marking pairs of matching parentheses inside-out and left-to-right, until all parentheses have been marked in which case it accepts the string. Otherwise, the string is rejected. The figure illustrates the program and its operation. (*a*) Alphabet molecules are considered one unit wide. They have a side-group representing the symbol, and left and right links for forming the tape polymer. (*b*) Transition molecules are two units wide and two units high. The molecule shown implements the left transition abbreviated *(,S1 → S0,#* and read: *If the control state is S1 and the head reads symbol ‘(’ to the left, then change state to S0, write symbol #, and move left one cell.* The molecule has a recognition site for the symbol ‘(’ and the state *S1* on its lower side, a side-group representing the new state *S0* above and a missing upper-right quadrant that accommodates the new symbol to be written, *#,* as well as left and right links enabling it to be part of the tape polymer. (*c*) These eight transition molecules shown schematically constitute the parenthesis checker program. The top row includes right transition molecules, which are read similarly to a left transition molecule (see (*b*)) with ‘right’ replacing ‘left’ through the description. The bottom row includes left transition molecules. The last transition enters into state *S3* and accepts the string*. Blank* recognizes the end of the non-blank part of the tape, namely the case where the transition molecule is at the end of the tape polymer. (*d*) Example transition that occurs during a computation on the string ‘(()()())’. The configuration consists of a tape polymer (1) and a trace polymer (2). An incoming right transition molecule *S0,) → #,S1* (3) loaded with an alphabet molecule # (4) that matches the current state of the current active molecule (5) and the alphabet symbol to its right (6). The updated configuration shows the displaced transition molecule (5) and displaced alphabet molecule (6) that are now part of the elongated trace polymer (2), with the incoming transition molecule (3), now the active transition molecule and the incoming alphabet molecule (4), both form part of updated the tape polymer (1).

The mechanical computer employs a chain of basic building blocks (figure 1*a*), referred to as *alphabet monomers*, to represent the Turing machine's tape, and uses another set of building blocks (figure 1*b*), referred to as *transition molecules*, to encode the machine's transition rules. The transition encoding is similar to a Wang [7] tile construction, which is also at the basis of DNA computing via self-assembly [8], and also to the concept of modified tRNA proposed as part of a ribosome-like computing device [9]. A transition molecule loaded with an alphabet monomer specifies a computational step of the computer similar to the way an aminoacyl-tRNA specifies a translation step of the ribosome [10]. The set of loaded transition molecules constitutes the computer's *program* (figure 1*c*).

The computer operates on two chains of building blocks simultaneously (figure 1*d*). One chain, referred to as the *tape polymer*, represents the Turing machine's tape and is edited by the computer similar to the way a Turing machine modifies its tape. The other chain, referred to as the *trace polymer*, is a by-product of the computation constructed incrementally from displaced transition molecules and displaced alphabet monomers, and has no analogue in the theoretical Turing machine. A transition molecule, referred to as the *active transition molecule*, joins the two polymers. The active transition molecule is embedded in the tape polymer and represents the location of the Turing machine's read/write head as well as the machine's internal state. At the same time, the active transition molecule is the terminal molecule of the trace polymer, representing the most recent transition of the computation. (Note that in this design, the read/write head is located between adjacent tape cells, not on a specific cell, unlike a standard Turing machine [1]; this change does not affect the computational capabilities of the machine.)

**Figure 2.**
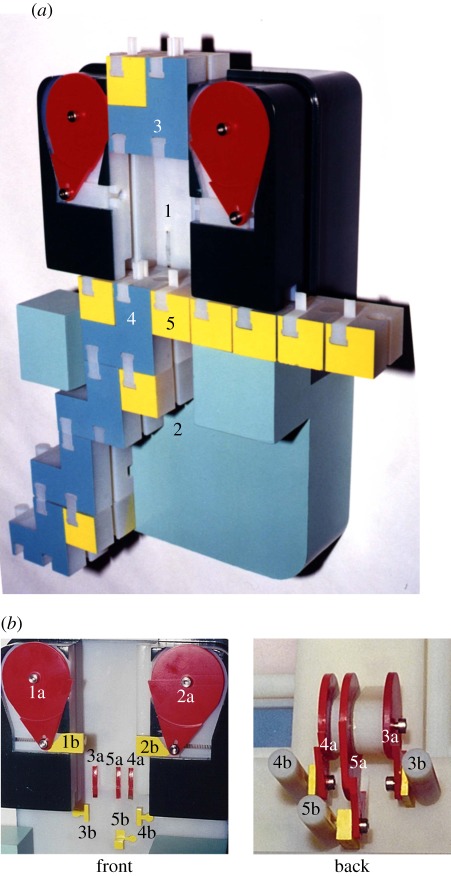
Mechanical computer. (*a*) The computer is 18 × 29 × 9 cm. The small tunnel (1) is part of the small subunit and is two units wide. The large tunnel (2) is part of the large subunit and is three units wide, so that it can accommodate the displaced transition molecule and the new active transition molecules. The small and large subunits can move one unit sideways relative to each other. Such movement is necessary following a change of direction of the computation. An incoming transition molecule (3) is approaching the active transition molecule (4) and the alphabet molecule to its right (5). The tape polymer can move left or right one unit, aligning the active transition molecule to the left or to the right side of the large tunnel. Such movement is necessary to accommodate consecutive transitions in the same direction. (*b*) Five mechanisms in the small tunnel prevent erroneous transitions from occurring. All mechanisms are based on a spring-loaded bellcrank/cam (a) which is connected to a linkage (b) which in its free state blocks passage of the approaching transition molecule. Each bellcrank/cam checks for a certain condition and if the condition is met, then is rotated. The connected linkage then moves out of the way of the approaching transition molecule, essentially effecting a conformational change in the tunnel. Two mechanisms (1, 2) detect that the (left or right) transition molecule is loaded with an alphabet molecule. Two mechanisms (3, 4) detect that a *Blank* recognition site matches the (left or right) end of the tape polymer and one mechanism (5) detects that the recognition site of the incoming transition molecule matches the state side-group of the active transition molecule and the alphabet symbol to its right. The computer was designed using SolidWorks Corporation's SolidWorks 98 software, and was manufactured on a 3D Systems Inc. SLA-5000 Stereolithography Apparatus by Scicon Technologies of Valencia, CA, USA. Material is a Dupont 8110 epoxy/polypropylene/polyethylene blend.

The computer (figure 2*a*) is made of two subunits, referred to as *small* and *large*, each with a tunnel called the *small tunnel* and the *large tunnel*, respectively. The small tunnel provides incoming loaded transition molecules with access to the active transition molecule and to its adjacent alphabet monomer. Access is controlled by gating mechanisms (figure 2*b*) which block transition molecules that are ill-formed or do not match the current state and current tape symbol. These mechanical analogues of allosteric conformational changes open the channel only when a valid incoming transition molecule approaches. The large tunnel holds the active transition molecule and the tail of the trace polymer being constructed.

The computer operates in cycles, processing one transition molecule per cycle. In each cycle, an incoming loaded transition molecule that matches the current state and its adjacent alphabet monomer becomes the new active transition molecule and its accompanying alphabet monomer is incorporated into the tape polymer. This is achieved by displacing the currently active transition molecule and the matched alphabet monomer, effectively editing the tape polymer, and elongating the trace polymer by the displaced molecules (figure 1*d*). Specifically, when processing a left transition molecule the computer moves left to accommodate the molecule, if necessary, and displaces the currently active transition molecule and the alphabet monomer to its left by the new molecule. The computer processes a right transition molecule similarly by moving right and displacing the alphabet monomer to the right of the active transition molecule. The theoretical Turing machine has an infinite tape, with only a finite portion of it being non-blank at any point during the computation. For obvious reasons, and in line with natural information representation by biopolymers, the mechanical Turing machine represents the two infinite blank portions of the tape implicitly. A special mechanism, shown in figure 2, detects the left and right ends of tape and treats each as a blank symbol. Special left blank transition molecules detect if the state they specify is at the left end of the tape and if so write a symbol and move to the left by activating this mechanism. Right blank transition molecules achieve the symmetric effect. The size of the mechanical Turing machine and its components do not make it susceptible to Brownian motion. Hence, assembly of transition molecules, pushing transition molecules down the small tunnel, and moving the small as well as large subunits relative to each other and relative to the tape polymer, all need to be carried out manually. The small as well as large units are designed and connected so that the small unit can wobble one symbol to the left or to the right relative to the large unit. In the left position, the current state and the symbol to its left are exposed to incoming left move transitions. Similarly, in the right position, right-move transitions may take effect. A peculiar aspect of the design is that this non-deterministic wobble precedes and enables the application of a corresponding move transition, and the transition taken has the effect of moving the wobble range one symbol in its direction (left or right). The computer is designed to be robust to Brownian motion in that only a transition which matches the current state and symbol can release the levers that would allow it to take effect.

When considering a future biomolecular realization of the mechanical Turing machine, one must realize that the device was designed to operate on three-dimensional building blocks by applying mechanical analogues of polymer elongation, cleavage and ligation, movement along a polymer, and control by molecular recognition unleashing allosteric conformational changes. Logically, the device is not more complicated than biomolecular machines of the living cell, and all its operations are part of the standard repertoire of these machines; hence, a biomolecular embodiment of the device is not infeasible. Specifically, a transition can be effected through the Brownian motion of an applicable loaded transition molecule into the tunnel of the small unit, followed by molecular recognition between the current state and symbol, and the state and symbol of the loaded transition molecule that triggers an allosteric conformation change. The conformational change in turn enables the incorporation of the new state and symbol instead of the old state and symbol, presumably through two cleavages and two ligations of the tape polymer.

The mechanical computer is similar to the ribosome in several other respects. Both operate on two polymers simultaneously, and their basic cycle consists of processing an incoming molecule that matches the currently held molecules on the first polymer, elongating the second polymer and moving sideways. Like the ribosome in the living cell, the computer requires supporting devices similar in function to aminoacyl-tRNA synthetases to load bare transition molecules with correct alphabet monomers, and a device similar in function to proteases to decompose the trace polymer and make its components available for reuse. However, unlike the ribosome, which only ‘reads’ the messenger RNA in one direction, the computer edits the tape polymer and may move in either direction.

The trace polymer created during the computation represents past state changes and head movements, as well as the symbols that were ‘erased’ from the tape during each transition, and as such has several important advantages. First, the trace polymer renders the computer reversible. Bennett [6] claims that, owing to thermodynamic considerations, von Neumann electronic computers are inherently energy-inefficient because their basic ‘store to memory’ operation irreversibly erases the content of the memory location. To remedy this inefficiency, Bennett proposed reversible computing, and in this context described a ‘hypothetical enzymatic Turing machine’. This hypothetical device is similar to our computer in representing the Turing machine's tape as a polymer of basic building blocks and in being dependent on the ‘Brownian motion’ of its building blocks to effect a computation. Because the trace polymer of the mechanical Turing machine embodies a complete record of the computation, a molecular implementation of the computer will be subject to the speed/energy consumption tradeoff of reversible devices. Furthermore, computation traces, in general, and the trace polymer, in particular, enable many ‘software’ program analysis and debugging tools [11], which are critically needed for large-scale applications. In addition, the trace polymer enables ‘hardware’ error detection and correction. One expects that any biomolecular implementation of the computer may exhibit a non-negligible error rate. By cascading computers along the same trace polymer, errors produced by one computer can be detected, and possibly also corrected, by its successor.

Perhaps the most important property of the mechanical computer is that it is reactive [12]: it can have an ongoing, program-controlled, interaction with its environment. This capability is a result of the biologically inspired architecture of the computer rather than inherited from the theoretical Turing machine, which was conceived as a ‘batch’ computing device that receives its input at the beginning of the computation and produces an output if and when the computation ends. The ribosome, for example, suspends the construction of a polypeptide chain when a required amino acid is unavailable. Similarly, our computer can be ‘programmed’ to suspend until a specific molecule is available. The availability of such a control molecule can be tied to other relevant environmental conditions, thus triggering a computation only when these conditions prevail.

The Turing machine is a non-deterministic computing device [1] in that it can make choices during a computation, and so is our computer. Not only it can have left and right transitions applicable simultaneously, but also it can have two or more left (or right) transitions with the same recognition site but with different target states or new symbols to be written. In a biomolecular implementation, this capability can be used to have the environment affect the course of a computation, based on the relative concentrations of molecules that enable one computational step compared with molecules enabling a different computational step. Using these two capabilities, the computer can be programed so that both the timing and the course of a computation are affected and controlled by the biochemical environment.

We endow the computer with an output device as follows. A simple extension to the Turing machine design is an instruction that erases the tape segment to the right of the read/write head. This instruction does not change the computing power of the machine, and for the theoretical model does not seem useful either. However, we interpret this instruction in our context to mean: ‘cleave the tape polymer to the right of the active transition molecule and release this tape polymer segment to the environment’. With this instruction, the computer can create and release any effectively computable polymer of alphabet monomers, in any number of copies, in the course of a computation. A cleaved tape polymer segment released by one computer can serve as the initial tape for the computation of another computer, or it can be ligated under certain conditions to the tape of another computer, thus enabling parallel processing, communication and synchronization among multiple operating computers.

## 3. Discussion

The computer design allows it to respond to the availability and to the relative concentrations of specific molecules in its environment, and to construct program-defined polymers as well as release them into the environment. Hence, if implemented using biomolecules, then the computer can be part of biochemical pathways. In particular, given a biomolecular implementation of the computer that uses ribonucleic acids as alphabet monomers, one can envision how cleaved tape polymer segments can function as messenger RNA, effecting program-directed synthesis of proteins in response to specific biochemical conditions within the cell. Such an implementation can provide a family of computing devices with broad biological and pharmaceutical applications.

## 4. Postscript

The mechanical Turing machine presented above was designed, constructed, written up and patented [13] in 1998, and presented in the fifth International Meeting on DNA-based computers in MIT, Boston (MA, USA) on 14–15 June 1999 [14]. The presentation included a slide, titled ‘Medicine in 2020’, showing a hypothetical biomolecular computer operating inside a living cell, sensing molecular disease symptoms and releasing a drug molecule in response. The slide, dubbed as ‘Doctor in a Cell’, drew the criticism of being over optimistic and was subsequently revised to ‘Medicine in 2050’ squelching any further criticism. Contemporary articulation of the vision of DNA computing being the basis of future smart drugs include the works of Cox *et al*. and Yurke *et al*. [15,16].

Subsequent attempts to publish the paper were not successful, so it was placed in the drawer for more than a decade; this paper, excluding §4, is essentially the shelved paper with some added explanations and references to address this journal's editor and reviewers comments. During that decade, much progress has been made in biomolecular computing towards the vision—outlined by this paper—of autonomous, programmable biomolecular computing devices capable of interacting with the biological environment [17–29], as well as in related directions [23,27,28,30–33], incorporating earlier conceptual work on molecular Turing machines [32,34] and advanced in synthetic biology [35,36]. In particular, the prediction that an autonomous, programmable molecular computing device may ‘compute synthetic biopolymers and release them into its environment in response to input from the environment’ is now a reality [19,29]. The realization that such a capability ‘may have broad pharmaceutical and biological applications’ being the basis of a new type of drugs is now a central tenet of the field of biomolecular computing, supplanting the initial noble, but apparently misguided, goal of beating electronic computers in their own game.

The Turing machine was perceived for decades as a theoretical widget devoid of practical relevance, especially given the overwhelming success of its younger alternative, the von Neumann stored-program computer architecture [2]. However, as our understanding of molecular cell biology and biochemistry unfolded, it became ever clearer that the concepts underlying the Turing machine are deeply rooted in nature. The Turing machine infinite tape, in which each cell may store one symbol taken from a finite alphabet, cannot be more similar, mathematically, to DNA, a potentially unbounded polymer in which each monomer is one of four letters. Molecular machines such as DNA polymerase, RNA polymerase and the ribosome are most naturally understood as simple finite-state transducers, a special case of the Turing machine.

A full-fledged realization of a biomolecular Turing machine according to the blueprint presented above, or a different one, is still a fairly distant reality, as much progress has to be made in protein and enzyme engineering before the necessary biomolecular building blocks can be fabricated to order and enable the realization of such a design. However, one can imagine that several decades hence, perhaps in an iGEM (International Genetically Engineered Machine)-like competition celebrating the 150th Turing anniversary, teams of students will be given the blueprint described in this paper and will be asked to realize it with their available tools. The ‘acid test’ of this paper would be whether these students would need further explanations or details to go about this task beyond what's shown in the paper. We argued that the mechanical Turing machine is in effect a functional specification for a biomolecular implementation, and we will ultimately be proved correct by the vote of such future students. As specifications and implementations go, we expect that with a rich enough biomolecular toolbox, many valid molecular implementations of this specification would be possible, the more the merrier.
